# Who lives in care homes in Greenland? A nationwide survey of demographics, functional level, medication use and comorbidities

**DOI:** 10.1186/s12877-021-02442-0

**Published:** 2021-09-18

**Authors:** N Albertsen, TM Olsen, TG Sommer, A Prischl, H Kallerup, S Andersen

**Affiliations:** 1 Department of Geriatric Medicine, Aalborg University Hospital, Aalborg, Denmark; 2 Arctic Health Research Centre, Aalborg University Hospital, Aalborg, Denmark; 3 Department of Clinical Medicine, Aalborg University, Aalborg, Denmark; 4 Department of Psychiatry, Regionalshospitalet Randers, Randers, Denmark; 5 Department of Anesthesiology and Intensive Care Medicine, Skåne University Hospital, Malmö, Sweden; 6 Department of Pathology, Odense University Hospital, Odense, Denmark; 7 Upernavik Health Center, Upernavik, Greenland; 8 Department of Internal Medicine, Queen Ingrid’s Hospital, Nuuk, Greenland; 9 Greenland Center for Health Research, Ilisimatusarfik, University of Greenland, Nuuk, Greenland

**Keywords:** Arctic, Greenland, Elderly, Care Homes, Inuit, Barthel Index, ADL, Polypharmacy, Inequality, Geriatrics

## Abstract

**Background:**

Greenland is facing an ageing population, and little is known about the characteristics of the elderly population in Greenland. This study offers both a comparison and a description of the demographics, causes of admission, comorbidities and medication of the residents in care homes in the capital, major and minor towns in four of the five administrative regions of Greenland.

**Methods:**

The study was conducted from 2010 to 2016 as a descriptive questionnaire-based cross-sectional study. Data from eligible residents from eight care homes were collected from the regular care staff. Data were categorised into three groups based on town size for analysis.

**Results:**

244 (100 %) of eligible residents participated in the study. Nearly 100 % were of Greenlandic ethnicity based on parents’ place of birth, and 62 % were women. The median age at admission/study was 69/71 years for men and 77/79 years for women (both *p* = 0.001). The median Body Mass Index was 25.6 kg/m^2^, more than half of the population were previous- or never-smokers and less than ten per cent consumed more than ten drinks of alcohol per week. The most common causes of admission were dementia (25.4 %), stroke (19.3 %) and social causes (11.1 %), while stroke (30.7 %), dementia (29.5 %) and musculoskeletal diseases (25.8 %) were the most common diagnoses at the time of the study. The Barthel Index was used to estimate the residents’ level of independence, and residents in smaller towns were found to have a higher level of independence than residents in the capital. The median number of prescribed medications was five, and more residents in the capital were prescribed more than ten medications than elsewhere in Greenland.

**Conclusions:**

This study is the first to describe care home residents in Greenland. We found a population younger than residents in comparable Danish care homes and that women were older than men at admission. In addition, care home residents in the capital had a lower level of independence and a higher number of prescribed medications, which could relate to differences in morbidity, access to health care services and differences in social circumstances influencing the threshold for care home admission.

**Supplementary Information:**

The online version contains supplementary material available at 10.1186/s12877-021-02442-0.

## Background

The world is ageing. According to the World Health Organization, worldwide life expectancy has increased five years between 2000 and 2015 [1], and by 2025, Earth will host more than 800 million people aged 65 + years [2].

The life expectancy of the Greenlandic people has increased from 58.0 years to 67.4 years for men and from 66.9 years to 72.7 years for women from 1977 to 2019 [3]. Furthermore, forecasts predict that the number of 70 + year-olds is expected to increase from 2,745 persons to 4,852 persons from 2020 to 2050 - an increase of 77 % among the oldest part of the population [4]. This rise emphasizes the need to describe and monitor the ageing population in Greenland.

There is a high occurrence of osteoporosis but a low treatment rate of the disease in Greenland [5], which suggests a higher risk of disability and therefore limited functional ability among the elderly. This is further supported by the population survey in Greenland from 2018, in which the Greenlandic elderly performed poorer at the chair stand test and the handgrip strength test compared to the elderly in the US and Germany [6].

A systematic description of the functional ability, comorbidities and medical treatment of the elderly in Greenland is lacking. This study aims to provide such a description focusing on the residents of care homes in Greenland. In addition, this study aims to assess whether these characteristics differ between residents of care homes in the capital, major and minor towns.

## Design and methods

### Settings and participants

**Fig. 1:**
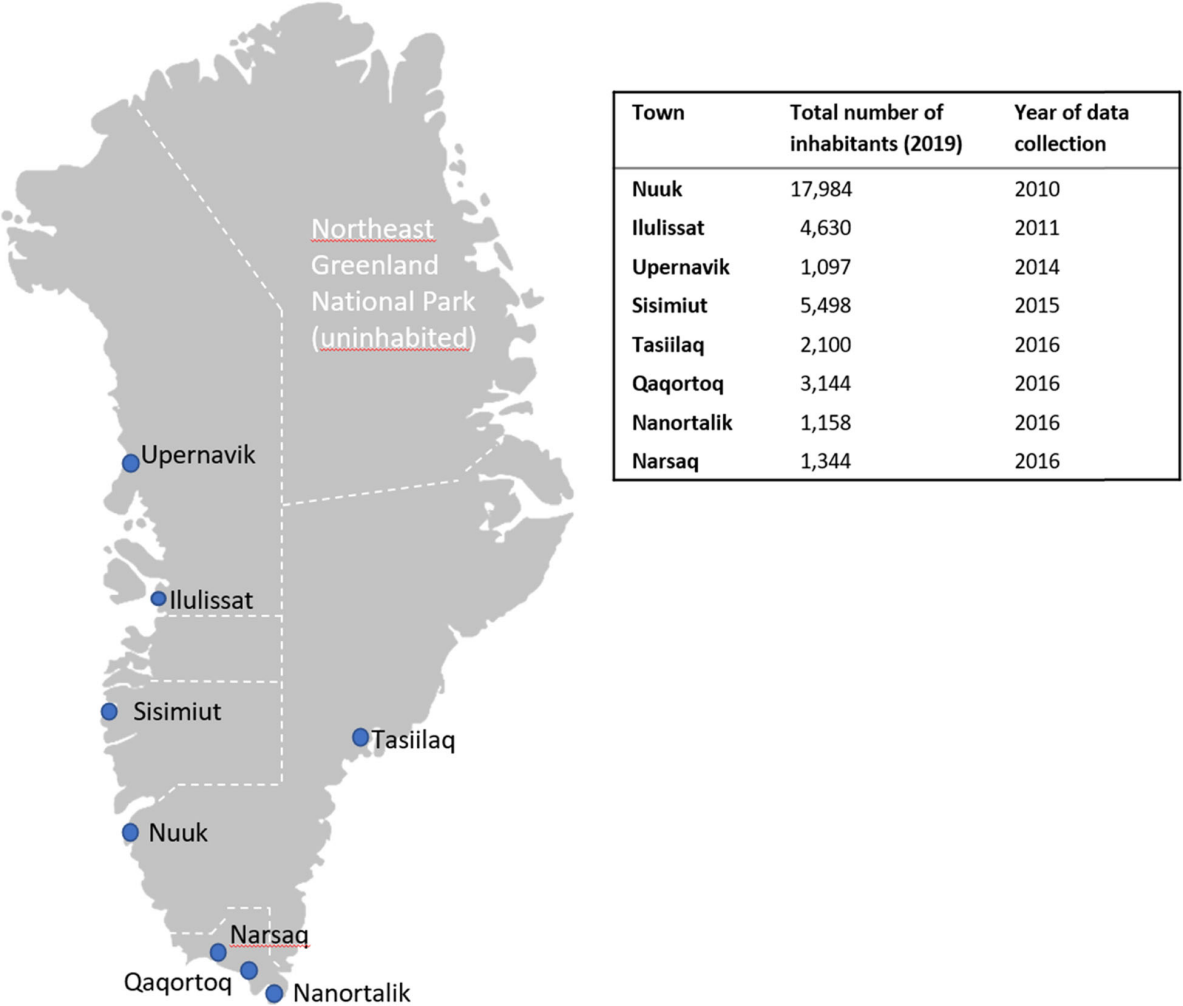
Locations of visited care homes and year of data collection. Greenland’s administrative regions are marked with white stapled lines (Fig. 1 is created in Microsoft PowerPoint v. 2105)

Greenland is the world’s largest island but sparsely populated, with only 56,000 inhabitants living in towns and settlements along the coast [7]. Greenland is divided into five administrative regions (Fig. 1), each with a chief medical officer responsible for the health care services in that specific region [8].

Due to a lack of infrastructure, access to advanced healthcare and diagnostics is heterogeneous and costly. The hospital in the capital, Nuuk, offers specialized forms of diagnostics and care such as MR scans and some forms of cancer treatment and more advanced diagnostics or care are possible by transfer to either Iceland or Denmark. Outside the capital, all larger towns have health care centres offering in-hospital treatment such as IV antibiotics and minor surgery. Smaller towns and settlements usually have a health care station operated by a nurse or a health care worker [8]. In case of medical emergencies, patients often need to be transported from smaller to larger towns [8].

The health care system in Greenland offers free health care to all residents with a permanent address in Greenland [8], including free prescription medicine and hospitalization. However, residency in a care home in Greenland is partly paid by the resident as 80 % of the resident’s retirement pension is deducted by the state as payment [9].

Approximately 14 % of the population surveyed in the population study in 2018 aged 55 years or older [6] lived in one of 18 care homes [9] in Greenland.

The study was conducted using questionnaire-based interviews in a cross-sectional study over a period of seven years (2010–2016).

Questionnaire-based interviews were conducted in the care homes of eight towns in Greenland: Nuuk, Ilulissat, Upernavik, Sisimiut, Tasiilaq, Qaqortoq, Nanortalik and Narsaq (Fig. 1). These care homes were selected based on accessibility and to represent towns of different sizes and locations in Greenland.

The inclusion criterium for participation was a residency in the care home at the time of the study. There were no exclusion criteria.

The care homes were visited in turn by a member of the research team (AP, HK, NA, SA, TO, TS). A questionnaire for each participant was filled out by a member of the regular nursing staff in collaboration with a research team member. The questionnaires were filled out based on the nursing staff’s knowledge of the resident and the information on the cause of stay, comorbidities, medication, height and weight stated in the residents’ charts in the care homes. In all but one home, no direct contact was made to the participants, and following the approval from the Greenlandic ethics committee, informed consent was waived in these places. In the remaining home, the residents participated in the filling out of questionnaires alongside the regular staff, but only after informed consent was obtained in Greenlandic. This was approved by the ethics committee in Greenland.

### Questionnaire and measurements

The questionnaire was developed, written and filled out in Danish, as Danish is the first language of the author initiating the study (SA) and of four of the five other authors (NA, TG, TO, HK). Furthermore, Danish is an official language of Greenland and spoken well by at least 70 % of the population [10].

The questionnaires included date of birth, age at study time, gender, height, weight, ethnicity (registered as Greenlandic if at least one parent was born in Greenland), length of stay at time of study, smoking status, alcohol consumption, history of goitre or thyroid disease, previous or current treatment with steroid or oestrogen, medical history, name and type of current medication and Barthel Index (BI) (Appendix 1).

BI comprises ten activities of daily life (ADL) concerning bladder and bowel control, feeding, grooming, transfers, bathing and mobility. The score is useful when assessing the participants’ independence, and a high score corresponds with a high degree of independence in ADL. The original BI was constructed in English by Dorothea Barthel in 1958 and later modified [11] [12]. Barthel 20, a modification made by Collin and Wade in 1988 [13], was used in this study in a Danish version based on the translation of Barthel 20 made by Maribo et al. in 2006 [12]. There is currently no Greenlandic version of Barthel 20, and the version used in this study is applied at the Department of Geriatric Medicine at Aalborg University Hospital.

The participants’ age at the time of admission was calculated using age and length of stay at the time of the study. Body Mass Index (BMI) was calculated using weight in kilogram divided by height in metres squared.

The questionnaire did not provide information on the date or cause of prescription for the stated medications, doses or regularity. All stated medications in the questionnaire were therefore counted towards the total number of drugs prescribed for each resident in this study. Cause of stay and comorbidities stated in the questionnaires were grouped by type for analysis: dementia, endocrine disease (diabetes, osteoporosis, thyroid disease, other), stroke, mental illness (depression, schizophrenia, other), neurological disease, pulmonary disease, cardiovascular disease (arrhythmia, other), urogenital disease, gastrointestinal disease, musculoskeletal disease, cancer, eye disease, other and unknown/not stated. “Old age”, “falling” and “social causes” were added as groups to cause of admission. The latter group includes residents who had either “social cause”, “unable to take care of themselves in their own home” or “admitted as an accompanying spouse” registered as the cause of admission on the questionnaire. Groups comprising more than five per cent of the total number of participants were included in the final analysis.

For analysis, age at admission was divided into six groups: younger than 65 years, age between 65 and 69 years, 70 and 74 years, 75 and 79 years, 80 and 84 years and 85 years or older. The total number of medications was grouped as nil, one to four, five to nine or ten medications or more. Polypharmacy was defined as five or more prescribed medications [14]. Finally, as a BI-score of nil corresponds to total dependency and a BI-score of 20 to total independency, these were also grouped for analysis.

 Place of residence was categorised into three groups to ensure the anonymity of the participants and allow us to compare residents from towns of different sizes. “Minor towns” were defined as having less than 2,000 inhabitants and include Nanortalik, Narsaq and Upernavik, “major towns” was defined as having more than 2,000 inhabitants and include Qaqortoq, Sisimiut, Tasiilaq and Ilulissat. Finally, the “capital” included only Nuuk as it is by far the largest town and offers the most extensive health care services [8].

### Statistics

All data were entered in EpiData v4.6.0.2 (the EpiData Association, Denmark) and analysed in STATA/MP16 (StataCorp LLC, USA).

Group characteristics were described using medians and interquartile range (IQR). Proportions were given in percentages.

Wilcoxon Rank Sum (non-categorical data) and Pearson´s chi-squared test (categorical data) were used to test for statistical difference between two groups, and Kruskal-Wallis Rank Test to test for statistical differences in medians between more than two groups. A p-value of less than 0.05 was considered statistically significant.

## Results

A total of 244 residents was included (100 % of residents eligible for participation). Approximately half of the questionnaires (46.3 %) were fully completed, and less than 12 % were missing more than three variables. Details of completeness and missing data can be found in appendix 2.

### Age at admission and time of study and length of stay

The detailed characteristics are given in Table 1. Women were markedly older than men at admission both nationwide (*p* = 0.001) and in the capital and major towns (*p* = 0.001 and *p* = 0.003). More residents were younger than 65 years at admission in the capital compared to major towns (*p* = 0.026).

**Table 1:** Characteristics of residents in care homes in Greenland

	Capital	Major Towns	Minor towns	Total
Number of residents	64	102	78	244
Gender (%), males	26 (40.6)	33 (32.4)	33 (42.3)	92 (37.7)
Age at time of study, years Median (IQR)	75.5 (66;82.5)	78 (71;82)	77 (71;83)	77 (70;82)
*Males*	66 (62;74)	72 (68;76.5)	75 (69;83)	71 (66;77)
* Females*	80 (74;84) ^e^**	80 (75;83) ^e^**	78 (74;83)	79.5 (74;84) ^d^**
Age at admission, years Median (IQR)	70 (63.5;78)	75 (68;80)	74 (67;79)	73.5 (66;79)
* Males*	65 (59;71)	69.5 (62;73.5)	70 (65;77)	69 (62;75)
* Females*	77.5 (69;81) ^e^**	78 (71;80) ^e^**	75 (69;79)	77 (70;80) ^d^**
Age at admission (%)				
* <65 years*	17 (26.6) ^a^*	13 (12.9)	13 (16.7)	43 (17.7)
* 65–69 years*	13 (20.3)	16 (15.8)	16 (20.5)	43 (17.7)
*70–74 years*	8 (12.5)	18 (17.8)	14 (18.0)	40 (16.5)
*75–79 years*	15 (23.4)	26 (25.7)	20 (25.6)	61 (25.1)
*80–84 years*	5 (7.8)	18 (17.8)	11 (14.1)	34 (14.0)
*85 + years*	6 (9.4)	10 (9.9)	6 (7.7)	22 (9.0)
Length of stay at time of study, months Median (IQR)	28 (12.5;55.0)	18 (12.0;47.0) ^c^*	48 (16.0;72.0)	26.5 (13.0;59.0)
BMI Median (IQR)	21.4 (14.0;28.7)	25.6 (20.7;32.8)	25.5 (21.9;30.1)	25.6 (21.3;31.0)
Mother born in Greenland (%)	No data	93 (97.9)	78 (100.0)	171 (98.3)
Father born in Greenland (%)	No data	92 (97.9)	77 (98.7)	169 (97.7)
Smoking (%), cigarettes per day				
*Never*	22 (34.9)	24 (24.7)	29 (37.7)	75 (31.7)
*Previous*	17 (27.0)	28 (28.9) ^c^*	11 (14.3)	56 (23.6)
*1–10*	10 (15.9)	34 (35.1) ^a^**	23 (29.9)	67 (28.3)
*11–19*	9 (14.3)	9 (9.3)	13 (16.9)	31 (13.1)
*20+*	5 (7.9)	2 (2.1)	1 (1.3)	8 (3.4)
Alcohol (%), drinks per week				
*Never*	19 (30.6) ^a^*^, b^*	49 (51.0)	40 (52.6)	108 (46.2)
*0–7*	35 (56.5)	39 (40.6)	31 (40.8)	105 (44.9)
*8–14*	7 (11.3)	7 (7.3)	4 (5.3)	18 (7.7)
*15–20*	1 (1.6)	1 (1.0)	0 (0.0)	2 (0.9)
* 21+*	0 (0.0)	0 (0.0)	1 (1.3)	1 (0.4)

*p < 0.05, **p < 0.01.

^a^: capital and major towns compared, ^b^: capital and minor towns compared, ^c^: major and minor towns compared, ^d^: all males and females compared, ^e^: males and females from the same town size compared.

Women were significantly older than men at the time of study in both the capital and major towns (*p* = 0.001 and *p* = 0.004). The residents in minor towns had stayed longer at the time of study compared to major towns (p = 0.01).

### Ethnicity, smoking and alcohol

An overall of 98 % of the participants in major and minor towns had at least one parent born in Greenland (Table 1). These data were missing from the capital.

Regarding smoking, more residents were previous smokers in major towns than in minor towns (*p* = 0.02), and a higher number of residents smoked between one and ten cigarettes per day in major towns compared to the capital (*p* = 0.008) (Table 1).

Almost half of the residents never consumed alcohol, and less than ten per cent consumed more than seven drinks per week (Table 1). A higher number of residents never consumed alcohol in both major and minor towns when compared to the capital (both *p* = 0.02).

### Cause of admission and comorbidities

The most common causes of admission recorded were dementia, stroke and social causes (Table 2). In the capital, neurological disease as a cause of admission was more common when compared to both major (*p* = 0.004) and minor towns (*p* = 0.01), and social causes and mental illness were more frequent causes of admission when compared to major towns only (*p* = 0.004 and 0.03).

**Table 2:** Cause of admission, comorbidity and Barthel Index

	Capital	Major towns	Minor towns	Total
Cause of admission (%)				
* ”Old age”*	0 (0.0)	11 (10.8) ^a^**	3 (3.9)	14 (5.7)
*Social causes*	12 (18.8) ^a^**	5 (4.9)	10 (12.8)	27 (11.1)
*Dementia*	14 (21.9)	32 (31.4)	16 (20.5)	62 (25.4)
*Stroke*	10 (15.6)	19 (18.6)	18 (23.1)	47 (19.3)
*Mental illness*	6 (9.4)	2 (2.0)	8 (10.3) ^c^*	16 (6.6)
*Neurological disease*	12 (18.8) ^a^ *^b^*	5 (4.9)	4 (5.1)	21 (8.6)
*Musculoskeletal disease*	6 (9.4)	5 (4.9)	12 (15.4) ^c^*	23 (9.4)
*Unknown/not stated*	2 (3.1)	21 (20.6) ^a^** ^c^**	3 (3.9)	26 (10.6)
Comorbidities (%)				
*Dementia*	20 (31.3)	33 (32.4)	19 (24.4)	72 (29.5)
*Neurological disease*	16 (25.0) ^a^* ^b^*	10 (9.8)	8 (10.3)	34 (13.9)
*Eye disease*	7 (10.9)	10 (9.8)	6 (7.7)	23 (9.4)
*Stroke*	26 (40.6)	27 (26.5)	22 (28.2)	75 (30.7)
*Cardiovascular disease*	10 (15.6)	22 (21.6)	21 (26.9)	53 (21.7)
*Pulmonary disease*	0 (0.0)	10 (9.8) ^a^*	12 (15.4) ^b^**	22 (9.0)
*Gastrointestinal disease*	0 (0.0)	12 (11.8) ^a^**	4 (5.1)	16 (6.6)
*Urogenital disease*	9 (14.1) ^a^*	4 (3.9)	6 (7.7)	19 (7.8)
*Musculoskeletal disease*	17 (26.6) ^a^*	20 (19.6)	26 (33.3) ^c^*	63 (25.8)
*Mental illness*	14 (21.9) ^a^*	9 (8.8)	10 (12.8)	33 (13.5)
- *Depression*	9 (14.1) ^a^*	5 (4.9)	7 (9.0)	21 (8.6)
*Endocrine disease*	13 (20.3)	10 (9.8)	13 (16.7)	36 (14.8)
- *Osteoporosis*	4 (6.3)	5 (4.9)	5 (6.4)	14 (5.7)
*Cancer*	3 (4.7)	6 (5.9)	5 (6.4)	14 (5.7)
*Other*	5 (7.8)	6 (5.9)	11(14.1)	22 (9.0)
*Unknown/not stated*	1 (1.6)	17 (16.7) ^a^** ^c^**	2 (2.6)	20 (8.2)
Barthel Index (0–20), Median (IQR)	13 (5;16)	12.5 (4;17)	14.5 (8;19) ^b^*	13 (6;17)
Full dependency (ADL 0) (%)	4 (6.3)	5 (4.9)	4 (5.2)	13 (5.3)
Full independency (ADL 20) (%)	2 (3.1) ^b^*	12 (11.8)	12 (15.4)	26 (10.7)

*p < 0.05, **p < 0.01.

^a^: capital and major towns compared, ^b^: capital and minor towns compared, ^c^: major and minor towns compared, ^d^: all males and females compared, ^e^: males and females from the same town size compared.

More residents had “old age” or “unknown/not stated” given as a cause of admission in major towns when compared to the capital (*p* = 0.007 and *p* = 0.002) and “unknown/not stated” when compared to minor towns (*p* = 0.02). In the minor towns, mental illness and musculoskeletal disease were more often stated as a cause of admission when compared to major towns (*p* = 0.01 and *p* = 0.017).

The most common diseases among the care home residents at the time of the study were stroke, dementia and musculoskeletal disease (Table 2). In the capital, neurological diseases were more common when compared to both major and minor towns (*p* = 0.009 and *p* = 0.02). In addition, more residents in the capital had a mental illness, depression or urogenital disease than in the major towns (all *p* = 0.02).

Residents from major towns more often had a gastrointestinal disease or a pulmonary disease than in the capital (*p* = 0.004 and *p* = 0.01), and more had “unknown/not stated” comorbidity in the major towns than in both the capital and minor towns (*p* = 0.002 and *p* = 0.02).

More residents in minor towns had musculoskeletal disease than in the major towns (*p* = 0.04), and pulmonary disease was more common than in the capital (*p* = 0.001).

### Barthel Index

Median BIs are listed in Table 2. The BI was higher in the minor towns than in the capital (p = 0.046). We found no difference in totally dependent (BI = 0) residents between towns. However, but the number of totally independent residents (BI = 20) was lower in the capital than in minor towns (*p* = 0.02) and may be lower in the capital than in the major towns as well (*p* = 0.051).

### Medication

The number of different medications per resident is detailed in Table 3. Participants in the capital were prescribed a higher number of medications compared to both major and minor towns (*p* = 0.001 and *p* = 0.002). Almost half of the residents were prescribed between five and nine medications and one third between one and four medications (Table 3). More residents in the capital were prescribed more than ten medications compared to both major and minor towns (both *p* = 0.001). In contrast, fewer were prescribed one to four medications in the capital than the rest of the country (*p* = 0.008 for major and *p* = 0.005 for minor towns).

**Table 3:** The number of medications prescribed in the capital, in major and in minor towns in Greenland

	Capital	Major towns	Minor towns	Total
Number of medications Median (IQR)	7 (5;10) ^a^** ^b^**	5 (3;8)	5 (3;7)	6 (3;8)
Number of medications (%)				
0	4 (6.3)	7 (6.9)	1 (1.3)	12 (4.9)
1–4	11 (17.2)	37 (36.3) ^a^**	30 (38.5) ^b^**	78 (32.0)
5–9	29 (45.3)	49 (48.0)	43 (55.1)	121 (49.6)
10+	20 (31.3) ^a^** ^b^**	9 (8.8)	4 (5.1)	33 (13.5)

**p < 0.01.

^a^: capital and major towns compared, ^b^: capital and minor towns compared, ^c^: major and minor towns compared, ^d^: all males and females compared, ^e^: males and females from the same town size compared.

## Discussion

This study is the first to describe the residents in Greenlandic care homes. As the study includes 100 % of eligible residents, of whom nearly all have Greenlandic ancestry based on parents’ place of birth, it also offers a unique knowledge of the elderly population in Greenland in general.

Overall, life expectancy for men and women in Greenland is s approximately ten years shorter than in Denmark [15]. Correspondingly, we found both men and women in our study to be nearly ten years younger than care home residents in Denmark both at the time of admission and the time of the study [16]. We furthermore found that only nine per cent of our study population were 85 years or older at the time of the study, compared to 54 % in the Danish study [16].

When comparing men and women, we found that women accounted for almost two-thirds of the study population and were eight years older than the men both at the time of admission and study. This eight-year difference contrasted with the five-year difference in life expectancy, as previous studies have indicated that increased age is equally associated with nursing home placement regardless of gender [17]. In accordance with our study, they found that admission rates were higher for women [17], which could be explained by the higher life expectancy of women [17] or social factors such as loneliness [18] or living alone [17], which has been shown to increase the risk of admission, although more so for men than women [17]. As our questionnaire did not include the social status of the residents before admission, possible differences between the genders in this regard was left unexplored in our study. Still, another study conducted among the general elderly population in Greenland describe suicidal thoughts and feelings of anxiety and unhappiness to be more common among women than men [6].

Regarding smoking, we found the proportion of never- and previous-smokers in our study to be 56 %, which is comparable to the general population of similar age in Greenland [19] but lower than in Denmark [20]. Pulmonary diseases were recorded among only nine per cent of our study population, suggesting that pulmonary disease is either underdiagnosed or less common compared to Denmark, where chronic obstructive pulmonary disease has been reported among 11 % per cent of the general population [21]. We also found that pulmonary diseases were reported more frequently in major and minor towns than in the capital, implying either higher diagnostic activity or actual higher prevalence.

As with smoking, alcohol consumption was markedly lower among the elderly in Greenland than in Denmark. Only nine per cent of our study population consumed more than seven drinks per week versus 31.3 % of the Danish population aged 65 years or older [20]. Our findings are similar to the self-reported consumption among the general population in Greenland of the same age [19]. The validity of our results is supported by the fact that less than five per cent of residents in our study had a cause of admission or comorbidity directly related to alcohol. However, some of the “social causes” for admission might include alcohol-related diseases.

The care home residents in our study also differed from the care home residents in Denmark regarding functional level. We found that five per cent of residents had a BI-score of zero, suggesting total dependency. This is ten times lower than the proportion found in Denmark [16]; however, we also found regional differences in Greenland as care home residents in minor towns had a higher median BI-score than in the capital and more totally independent residents with a BI-score of 20. This discrepancy could be explained by differences in local opportunities regarding general health care as iller patients are often transferred to the capital [8], and some elderly patients may choose to remain there. It could also be speculated that the capital offers more support for the elderly in their own homes, postponing care home residency longer than what is possible in smaller towns.

We came upon regional differences regarding causes of admission, comorbidities and the number of prescribed medications. Dementia, stroke and social causes were the most common causes of admission nationwide, although dementia was a less frequent cause in the capital than in major and minor towns. Neurological diseases were more commonly stated as both cause of admission and comorbidity in the capital, which may be explained by a higher diagnostic accuracy here than in the rest of Greenland, as CT- and MR-scanners are only available in the capital. This explanation is further supported by the fact that 21 % of the residents in the major towns had an “unknown/not stated” cause of admission, while this was only three per cent in the capital. Dementia among the elderly in Greenland also calls for further attention. Although we found dementia to be the second most common illness and diagnosed among 29.5 % of our study population, it seems to be underdiagnosed in Greenland compared to other countries. Dementia affected 45 % of the study population in Denmark [16] and 57.4 % of the population in a Scottish care home [22], and these results are in keeping with other studies that reported dementia to be common among indigenous people [23].

Finally, fewer residents in the capital were prescribed between one and four medications, and more were prescribed ten or more medications compared to both major and minor towns. This correlates to our finding fewer independent residents in the care home in the capital and could imply a higher number of comorbidities with parallel polypharmacy. The overall nationwide number of prescribed medications in our study was in accordance with findings by studies from other parts of the world, as almost 65 % of the residents were prescribed five or more medications. For comparison, a cross-sectional study of the Danish population aged 65 years or older found that 63 % of patients with dementia and 35 % of patients without dementia were prescribed at least five medications [24] and a Canadian study comprising 1,711 institutionalized elderly found current use of five or more medications among 53 % [25].

### Strengths and limitations

Strengths: This is the first description of the population of Greenlandic care homes on a national scale. The study covered four out of five regions and included eight out of 18 care homes in Greenland. All eligible residents were included at the surveyed care homes, and the questionnaires were completed by the staff who knew the residents. Furthermore, the same questionnaire and procedures were used in seven out of eight care homes, securing a high degree of uniformity of the collected data.

Limitations: Ideally, this study should have been conducted in Greenlandic. As stated in the methods section, the questionnaire was written and filled out in Danish, and this may have caused errors or omissions when filled out by a member of the care staff who speaks Greenlandic as his or her mother tongue. However, the number of native Greenlandic speakers who understand and speak Danish well has been rapidly increasing over the years [8], and Danish is the most commonly used language in most public workplaces [8]. Also, a member of the research staff was present and available for questions. Therefore, we believe that errors or omissions due to language difficulties have been kept to a minimum. Also, the care staff completing the questionnaires might have had varying experience with the BI, and the Barthel 20 has not, to the authors’ knowledge, been validated in Danish yet, although the version used in this study is used in everyday clinical practice in a university hospital in Denmark. Furthermore, the care homes were visited over a period of seven years. Any changes in the characteristics of the residents in care homes that may have taken place during this period are not included in this study but should be considered in future studies and publications.

Ethnicity was based on the birthplace of the participants’ parents as used elsewhere in the literature [26]. However, this may have overestimated the number of participants being of Inuit ethnicity. Finally, information about the care homes’ training facilities or utilities such as lifts were not included in the study, and these may influence the demographics of the residents. Recording of social parameters or hometown of the residents could also have been relevant and should be considered in future studies.

### Conclusion

This study provides a description of important traits of the population in care homes in Greenland and a comparison between towns of different sizes. Overall, we found low alcohol consumption rates, polypharmacy, suggestions of low diagnostic accessibility and a distinct gender difference in the study population. Moreover, regional differences in the population residing in care homes suggest inequality in access to healthcare services.

Knowledge of the elderly population is essential to accommodate the ongoing changes in the age composition in the society, and the welfare of the growing elderly population has gained increasing attention in Greenland [27]. Additional studies focusing on the aspects raised in this paper are warranted.

## Supplementary Information

**Additional file 1.**

**Additional file 2.**

**Additional file 3.**

## Data Availability

The dataset used during the current study is available from the corresponding author on reasonable request.

## Abbreviations

BI: Barthel Index
ADL: Activities of Daily Life
BMI: Body Mass Index
NSAID: Non-Steroid Anti-Inflammatory Drugs
PPI: Proton Pump Inhibitors

## References

[1] WHO: Life expectancy increased by 5 years since 2000, but health inequalities persist. http://www.who.int/mediacentre/news/releases/2016/health-inequalities-persist/en/ (Accessed 17 July 2020).

[2] WHO: The World Health Report 1998. Life in the 21st century - A vision for all (who.int) (Accessed 26 July 2021)

[3] Statistics Greenland. Middellevetid 5-års grundlag efter type, bosted, fødested, køn og tid. PxWeb (stat.gl) (Accessed 27 July 2021).

[4] Statistics Greenland. Befolkningsfremskrivning 2020 efter fremskrivning, område, alder, fødested og tid. PxWeb (stat.gl) (Accessed 27 July 2021)

[5] Jakobsen A, Laurberg P, Vestergaard P, Andersen S (2013). Clinical risk factors for osteoporosis are common among elderly people in Nuuk, Greenland. Int J Circumpolar Health.

[6] Nørtoft K, Bjerregaard P, Hounsgaard L et al. Ældre menneskers liv og helbred i Grønland. en rapport fra forsknings- og udviklingsprojektet Arktisk Aldring (AgeArc) [The life and health on the elderly in Greenland. A report from the research- and development project Arctic Aging (AgeArc). In Danish]. 2019. aeldre_menneskers_liv_og_helbred_i_groenland.pdf (sdu.dk) (Accessed 12 May 2021)

[7] Statistics Greenland. Befolkningen 1. januar efter bostedstype, fødested, køn og tid. PxWeb (stat.gl) (Accessed 27 July 2021).

[8] Niclasen B, Mulvad G. Health care and health care delivery in Greenland. Int J Circumpolar Health. 2010 Dec;69(5):437–47. 10.3402/ijch.v69i5.17691 21118636

[9] Naalakkersuisut. Vores velstand og velfærd - kræver handling nu. [Our prosperity and welfare - demand action now. In Danish] 2011. Microsoft Word - SVK Betænkning 110307 (naalakkersuisut.gl) (Accessed 12 May 2021)

[10] Frederiksen K, Olsen CC. Det grønlandske sprog i dag. [The Greenlandic language today. In Danish]. 2017. DET GRØNLANDSKE SPROG I DAG (naalakkersuisut.gl) (Accessed 12 May 2021).

[11] Sainsbury A, Seebass G, Bansal A, Young JB (2005). Reliability of the Barthel Index when used with older people. Age Ageing.

[12] Maribo T, Lauritsen JM, Waehrens E, Poulsen I, Hesselbo B. [Barthel Index for evaluation of function: a Danish consensus on its use. In Danish]. Ugeskr Laeger. 2006;168(34):2790–2. 16942697

[13] Collin C, Wade DT, Davies S, Horne V (1988). The Barthel ADL Index: a reliability study. Int Disabil Stud.

[14] Masnoon N, Shakib S, Kalisch Ellett L (2017). What is polypharmacy? A systematic review of definitions. BMC Geriatr.

[15] Statistics Denmark: populations and elections. https://www.dst.dk/en/Statistik/emner/befolkning-og-valg. (Accessed July 2020).

[16] Beck AM, Damkjær K, El Kholy K et al. Plejetyngden af ældre danskere på plejehjem. [The care requirements of residents in Danish nursing homes. In Danish). Ugeskr Laeger 2008;170 (9):749–52. 18307964

[17] Luppa M, Luck T, Weyerer S (2009). Gender differences in predictors of nursing home placement in the elderly: a systematic review. Int Psychogeriatr..

[18] Hanratty B, Stow D, Moore DC et al. Loneliness as a risk factor for care home admission in the English Longitudinal Study of Ageing. Age Ageing 2018; 47(6):896–900. 10.1093/ageing/afy095 PMC6481549 30007359

[19] Larsen CVL, Hansen CB, Ingemann C. Befolkningsundersøgelsen i Grønland 2018 - Levevilkår, livsstil og helbred. Oversigt over indikatorer for folkesundheden [The Greenlandic Population Study 2018. Living conditions, lifestyle, and health: Indicators for public health. In Danish] befolkningsundersoegelsen_i_groenland_2018_dansk.pdf (sdu.dk) (Last accessed Aug 2020)

[20] Jensen HAR, Davidsen M, Ekholm O et al. Danskernes Sundhed - Den Nationale Sundhedsprofil 2017. [The Health of the Danes - the national health profile 2017. In Danish]: https://www.sst.dk/da/udgivelser/2018/danskernes-sundhed-den-nationale-sundhedsprofil-2017 (Accessed Aug 2020).

[21] Çolak Y, Afzal S, Nordestgaard BG (2017). Prognosis of asymptomatic and symptomatic, undiagnosed COPD in the general population in Denmark: a prospective cohort study. Lancet Respir Med.

[22] Burton JK, Lynch E, Love S (2019). Who lives in Scotland’s care homes? Descriptive analysis using routinely collected social care data 2012–2016. J R Coll Physicians Edinb.

[23] Li SQ, Guthridge SL, Aratchige PE (2014). Dementia prevalence and incidence among the Indigenous and non-Indigenous populations of the Northern Territory. MJA.

[24] Kristensen RU, Nørgaard A, Jensen-Dahm C (2018). Polypharmacy and Potentially Inappropriate Medication in People with Dementia: A Nationwide Study. J Alzheimers Dis.

[25] Ramage-Morin PL. Medication use among senior Canadians. Statistics Canada. Catalogue no. 82-003-XPE. Health Reports, vol 20, no. 1. March 2009. 19388367

[26] Rex KF, Krarup HB, Laurberg P (2012). Population-based comparative epidemiological survey of hepatitis B, D, and C among Inuit migrated to Denmark and high endemic Greenland. Scand J Gastroenterol.

[27] Naalakkersuisut. Ældre i Fremtidens Grønland. Naalakkersuisuts ældrestrategi 2012–2015. [The Elderly in Future Greenland. The aging strategy of Naalakkersuisut 2012–2015. In Danish] http://www.ga.gl/LinkClick.aspx?fileticket=quIyVrBHQ7Q%3D&tabid=1816&language=da-DK. (Accessed 1 Aug 2020).

