# Supplementary material for: Who lives in care homes in Greenland? A nationwide survey of demographics, functional level, medication use and comorbidities
Source: BMC Geriatr. 2021 Sep 18;21:500. doi: 10.1186/s12877-021-02442-0 (PMC8449891; doi:10.1186/s12877-021-02442-0)
Supplement: Supplementary file 1 — Additional file 1. [file 12877_2021_2442_MOESM1_ESM.docx]

PARTICIPANT

1. Male □
2. Female □
3. Height ­­ cms
4. Weight kgs
5. Age years
6. Father born in Greenland Yes □ No □
7. Mother born in Greenland Yes □ No □

**2. Fractures** Yes □ No □ Type:

**3. Tobacco** – cigarettes: **4. Alcohol:**

1. Never □ 1. Never □
2. Previously □ 2. 0-7 drinks per week □
3. 1-10 □ 1. 8-14 drinks per week □
4. 11-20 □ 1. 15-21 drinks per week □
5. More than 20 □ 1. More than 21 drinks per week □

5. Previous job/education:

CARE:

1. Care home Arrival month: year:

1. Duration of stay:
2. Cause of stay:
3. Hours spent outside: □ <1 □ 1-3 □ > 4 hours per day

2. Supplementary **vitamins**:

1. Never □
2. Sometimes □
3. Daily □

If yes, do they contain:

1. **Iodine:** Yes □ No □ Don’t know □
2. **Vitamin D** Yes □ No □ Don’t know □
3. **Calcium** Yes □ No □ Don’t know □

HISTORY:

3. Do you have, or have you had, **goiter or thyroid disease**?

Yes, high □ Yes, low □ Yes, normal □ No □ Don’t know □

4. Other **diseases**:­

1. 19­­­­­­­__

2. 19­­­­­­­__

3. 19­­­­­­­__

4. 19­­­­­­­__

4. Are you prescribed any **medication**? Yes □ No □

1. 19­­­­­­­__

2. 19­­­­­­­__

3. 19­­­­­­­__

4. 19­­­­­­­__

Prednisolone: Yes □ No □ Dose/duration:

Hormone/Estrogen: Yes □ No □ Dose/duration:

Barthel

ADL index (BAI): **Resident:** Date of birth: **Study-number:**

| **FUNCTION** | **SCORE** | **DESCRIPTION OF WHAT HE/SHE DOES:** |
| --- | --- | --- |
| Bowels | 0  1  2 | Incontinent (or needs laxatives from personnel)  Occasional accident – maximum once per week  Continent |
| Bladder | 0  1  2 | Incontinent or catheterized and unable to manage without help  Occasional accident – maximum once per week  Continent (more than 7 days) |
| Grooming | 0  1 | Needs help for: toothbrushing, shaving, combing, washing face  Independent |
| Toilet Use | 0  1  2 | Dependent  Needs some assistance  Independent |
| Feeding | 0  1  2 | Dependent  Needs assistance cutting food, buttering bread etc.  Independent when food is put within reach |
| Transfer | 0  1  2  3 | Dependent – no sitting balance  Can sit, but requires physical assistance from 1-2 persons  Independent, but requires verbal guidance or assistance  Independent |
| Mobility:  Indoors | 0  1  2  3 | Immobile  Wheelchair independent  Walks with help from 1 person (verbal or physical)  Independent |
| Dressing | 0  1  2 | Dependent  Can do about half unaided  Independent |
| Stairs | 0  1  2 | Unable  Needs help  Able, independent up and down |
| Bathing | 0  1 | Dependent on some form of assistance  Independent |

**Total**
