# Supplementary material for: Who lives in care homes in Greenland? A nationwide survey of demographics, functional level, medication use and comorbidities
Source: BMC Geriatr. 2021 Sep 18;21:500. doi: 10.1186/s12877-021-02442-0 (PMC8449891; doi:10.1186/s12877-021-02442-0)
Supplement: Supplementary file 2 — Additional file 2. [file 12877_2021_2442_MOESM2_ESM.docx]

Appendix 2

| Variable | No. of participants missing data (%) |
| --- | --- |
| Gender | 0 (0) |
| Aget at time of study | 1 (0.4) |
| age at Admission | 0 (0) |
| length of stay | 0 (0) |
| Height | 117 (47.9) |
| weight | 43 (17.6) |
| Mother Born in Greenland | 70 (28.7) |
| father born in greenland | 71 (29.1) |
| smoking | 7 (2.9) |
| alcohol | 10 (4.1) |
| Cause of stay | 24 (9.8) |
| comorbidity | 0 (0) |
| number of drugs | 0 (0) |
| names of drugs | 0 (0) |
| adl | 0 (0) |
