# Supplementary material for: Who lives in care homes in Greenland? A nationwide survey of demographics, functional level, medication use and comorbidities
Source: BMC Geriatr. 2021 Sep 18;21:500. doi: 10.1186/s12877-021-02442-0 (PMC8449891; doi:10.1186/s12877-021-02442-0)
Supplement: Supplementary file 3 — Additional file 3. [file 12877_2021_2442_MOESM3_ESM.pdf]

ADL-vurdering i Grønland

2009-2010

Barthel

ADL index (BAI): Hvad gør **Beboer**: __________ Fødselsdato: __________ **NR** ______

FUNKTION	SCORE	BESKRIVELSE af hvad han/hun gør:
Tarm	0	Inkontinent for afføring / Brug for laxantia ved hjælp af personale.
	1	Uheld af og til - max 1 gang om ugen.
	2	Kontinent: Kan holde på afføringen.
Blære	0	Inkontinent eller har katheter, som passes af andre.
	1	Uheld af og til - max 1 gang om dagen.
	2	Kontinent (mere end 7 dage).
Personlig Hygiejne	0	Hjælp til: Tandbørstning, barbering, frisere sig, vaske ansigt.
	1	Kan selv, evt efter opfordring.
Toiletbesøg	0	Afhængig af hjælp (af- på, tøj ned-op, tørres).
	1	Kan noget selv, men har brug for lidt hjælp.
	2	Kan alt selv (af-på, tøj ned-op, tørre sig).
Spisning	0	Skal hjælpes med alt.
	1	Hjælp til: Skære i stykker, smørre brød, mv.
	2	Kan selv spise, når maden sættes frem.
Seng-til-stol	0	Kan intet - ikke balance til at sidde.
	1	Kan sidde, men kræver fysisk hjælp af 1 - 2 personer.
	2	Kan selv, men skal opfordres og forklares (verbal hjælp).
	3	Kan og gør selv - uafhængig af hjælp.
Mobilitet: Komme omkring indendørs	0	Immobil.
	1	Kørestol, klarer sig helt selv.
	2	Kan gå med hjælp fra 1 person (verbal eller fysisk).
	3	Kan selv - Uafhængig af hjælp.
Påklædning	0	Kan intet - Skal hjælpes med alt.
	1	Kan ca ½ selv. Hjælpes med f.eks. knapper, lynlås, snørrebånd.
	2	Kan selv - Uafhængig af hjælp.
Trappegang	0	Kan ikke.
	1	Kan noget, men skal have hjælp.
	2	Kan selv - Uafhængig af hjælp op og ned.
Bad	0	Kan ikke uden hjælp under en eller anden form.
	1	Kan alt selv - Uafhængig af hjælp.

SUM: ________

DELTAGER:

i. Mand ☐
ii. Kvinde ☐

b. Højde _________ cm
c. Vægt _________ kg
d. Alder _________ år
e. Far født på Grønland Ja ☐ Nej ☐
f. Mor født på Grønland Ja ☐ Nej ☐

2. **Knoglebrud**: Ja ☐ Nej ☐ Type: ____

3. Alder for sidste menstruation: _______ år

4. **Tobak** - Cigaretter**:**

a. aldrig ☐
b. holdt op ☐
c. 1-10 ☐
d. 11-20 ☐
e. flere end 20 ☐
f. ________________

5. **Alkohol**:

a. aldrig ☐
b. 0-7 genstande pr uge ☐
c. 8-14 genstande pr uge ☐
d. 15-21 genstande pr uge ☐
e. mere end 21 genstande pr uge ☐
f. __________________________

PLEJE:

1. Plejehjem - Ankomst Mdr. ______ År______

a. Varighed: ____________________
b. Årsag: _______________________
c. UDE ☐<1 ☐1-3 ☐>3 timer/dag

2. Får **Vitaminer**:

(1) Aldrig ☐
(2) Af og til ☐
(3) Daglig ☐

Hvis ja, indeholder de da

(a) **Jod**: Ja ☐ Nej ☐ Ved ikke ☐
(b) **D-vit** Ja ☐ Nej ☐ Ved ikke ☐
(c) **Calc**ium Ja ☐ Nej ☐ Ved ikke ☐

JOURNAL:

3. **Anden sygdom:** Ja ☐ Nej ☐

4. **Hvis ja, hvilke**:

i. 19__ _______________________________________
ii. 19__ _______________________________________
iii. 19__ _______________________________________
iv. 19__ _______________________________________

5. Får du **Medicin:** Ja ☐ Nej ☐

(1) __________________________
(2) __________________________
(3) __________________________
(4) __________________________

b. Prednisolon Ja ☐ Nej ☐ Dosis /Varighed:____________________
c. Hormon / Østrogen Ja ☐ Nej ☐ Dosis /Varighed:____________________
